# Socioeconomic inequality in organized and opportunistic screening for breast cancer: results from the Korean National Cancer Screening Survey, 2009-2021

**DOI:** 10.4178/epih.e2025031

**Published:** 2025-05-30

**Authors:** Yejin Ha, Xuan Quy Luu, Woorim Kim, Jae Kwan Jun, Mina Suh, Kui Son Choi

**Affiliations:** 1 National Cancer Control Institute, National Cancer Center, Goyang, Korea; 2 Department of Biostatistics, Faculty of Fundamental Science, Hanoi University of Public Health, Hanoi, Vietnam; 3 Graduate School of Cancer Science and Policy, National Cancer Center, Goyang, Korea

**Keywords:** Breast cancer, Mass screening, Health inequities, Korea

## Abstract

**OBJECTIVES:**

Breast cancer screening rates have increased since the introduction of the National Cancer Screening Program (NCSP) in Korea. However, it remains unclear whether socioeconomic inequalities have improved, particularly according to screening type. This study investigated inequalities in organized (government-led) and opportunistic (individually initiated) screening, stratified by education and income levels.

**METHODS:**

Data were obtained from the Korean National Cancer Screening Survey, conducted annually from 2009 to 2021, involving approximately 1,700 women each year except in 2009. Trends were analyzed using joinpoint regression to calculate average annual percent changes (AAPCs). Socioeconomic inequalities were assessed using the slope index of inequality (SII) and relative index of inequality (RII).

**RESULTS:**

Organized screening rates increased from 42.0% in 2009 to 60.2% in 2021 (AAPC, 1.9; 95% confidence interval [CI], 0.7 to 3.4), whereas opportunistic screening rates declined from 13.3% to 11.2% (AAPC, -5.4; 95% CI, -8.7 to -2.3). For organized screening, individuals with lower education levels exhibited higher participation, resulting in negative inequality indices (SII, -5.37%; RII, 0.80). No significant income-based inequality was found (SII, 1.60%; RII, 1.07). However, opportunistic screening demonstrated significant inequalities by both education (SII, 5.37%; RII, 1.92) and income (SII, 5.90%; RII, 1.96), with higher participation rates among more advantaged groups.

**CONCLUSIONS:**

The NCSP has improved breast cancer screening rates and reduced income-related inequality in organized screening. However, educational and income-based inequalities persist in opportunistic screening. To reduce screening inequities, policy efforts are needed to further promote the NCSP, including improving program quality and providing financial support for follow-up examinations.

## GRAPHICAL ABSTRACT

[Fig f4-epih-47-e2025031]

## Key Message

This study examined socioeconomic inequality in breast cancer screening in Korea by screening type. While organized screening has improved overall participation and reduced income-related inequality, socioeconomic inequalities remain in opportunistic screening.Continued and targeted policy efforts for the NCSP are necessary to further reduce inequality in breast cancer screening.

## INTRODUCTION

Breast cancer is the most frequently diagnosed cancer and the fifth leading cause of cancer-related mortality among women globally [1,2]. In 2020, an estimated 2.3 million new cases and 685,000 breast cancer-related deaths occurred, accounting for 1 in 4 cancer diagnoses and 1 in 6 cancer deaths worldwide. Breast cancer is the most prevalent malignancy in 159 countries and the leading cause of cancer mortality in 110 countries [1]. In Korea, breast cancer incidence has steadily increased over recent decades, and it has been the most common cancer among women since 2019 [3].

Breast cancer survival rates vary considerably across regions and countries. In most high-income countries, the 5-year survival rate exceeds 90%, whereas it is approximately 66% in 12 sub-Saharan African countries [4]. These disparities highlight global inequalities in breast cancer outcomes and emphasize the need for enhanced access to early detection and effective treatment worldwide.

In Korea, 2 types of breast cancer screening programs are available: organized and opportunistic screening. Organized screening refers to systematic, government-led programs offering screenings at regular intervals to eligible populations, typically at low or no cost. Organized cancer screening in Korea was initiated through the National Cancer Screening Program (NCSP) in 1999, aiming to increase screening rates, improve survival, and reduce cancer-related deaths through early detection [5,6]. Medical Aid recipients and lower-income beneficiaries of the National Health Insurance Service (NHIS) receive free screenings, while individuals in the upper 50% income bracket are required to pay a 10% out-of-pocket fee [7,8]. For breast cancer, the NCSP currently offers biennial mammography for women aged 40 years and older [9]. In contrast, opportunistic screening involves screenings initiated by healthcare providers or requested by individuals, with participants responsible for the associated costs [10,11]. Opportunistic screenings are fully paid for by the individual and may offer greater flexibility, shorter waiting periods, or additional diagnostic options.

Socioeconomic inequalities in cancer screening participation are well-documented. Education, income, and health-related background are strongly associated with participation rates [12,13]. In both Korea and other countries, lower socioeconomic status (SES) groups consistently demonstrate lower participation rates, contributing to disparities in breast cancer outcomes [9,14-17]. While some studies suggest organized screening programs can reduce these inequalities, others report persistent or even widening gaps. For example, a study conducted in Spain reported reduced inequalities following the introduction of an organized screening program [18]. In contrast, studies conducted in Korea have indicated that although the NCSP has improved overall breast cancer screening rates, income-related inequalities persist and, in some cases, have worsened [7,9]. This inconsistency may stem from the fact that most previous studies have either examined overall breast cancer screening rates or focused solely on organized screening without differentiating between organized and opportunistic types. Furthermore, research examining how the implementation of the NCSP influenced participation rates in both organized and opportunistic screening, and how these changes affected socioeconomic inequalities, remains limited. Therefore, a separate analysis of these 2 screening types is necessary to better understand their respective contributions to disparities in breast cancer screening. Additionally, the potential impact of the coronavirus disease 2019 (COVID-19) pandemic must be considered, as it may have altered screening behaviors by affecting healthcare accessibility and individual preferences.

Accordingly, this study assessed breast cancer screening rates and socioeconomic inequalities in organized and opportunistic screening in Korea, stratified by education and income levels. Based on the hypothesis that inequalities would be lower in organized screening and higher in opportunistic screening following the introduction of the NCSP, we examined trends and differences in participation rates across SES groups using inequality indices.

## MATERIALS AND METHODS

### Data source

Data were derived from the Korean National Cancer Screening Survey (KNCSS) from 2009 to 2021. The KNCSS is an annual, nationwide, cross-sectional survey conducted by the National Cancer Center since 2004. Study participants were selected using stratified, multistage random sampling based on resident registration population data. Enumeration districts were defined according to population size, stratified by gender, age, and geographic region. Within each selected district, households were randomly sampled using stratified sampling methods, involving 9 to 11 households in urban areas and 14 to 16 households in rural areas [19]. Survey sample weights, provided by the Population and Housing Census by Statistics Korea, were applied to ensure national representativeness [20]. Participants were recruited via door-to-door visits conducted by a professional research agency, with in-person interviews administered. At least 3 attempts were made to contact each household, and 1 individual per household was selected to optimize response rates [19,21].

The number of participants varied annually; for example, from 2009 to 2010 and from 2013 to 2014, the number of participants in the KNCSS increased from 2,000 to 4,056 and from 4,100 to 4,501, respectively. According to the NCSP protocol, the study population included women aged 40-74 years without a prior diagnosis of breast cancer. Approximately 1,700 women within this age group were eligible for breast cancer screening each year, except for 2009. A total of 21,974 participants were included in the final analysis.

### Screening classification

Study participants were asked about their socio-demographic characteristics and breast cancer screening history. Screening history was assessed using the following questions: “Have you ever undergone breast cancer screening?”, “Which screening method have you experienced?”, “When was your most recent screening with this method?”, and “What was your payment method for breast cancer screening?” Screening status was defined as “screened” for women who underwent mammography within the previous 2 years, in line with the Korean National Cancer Screening Guideline. Women were categorized as “non-screened” if they had never undergone screening, if their most recent mammography was more than 2 years ago, or if they had undergone only an ultrasound test, which is excluded from the NCSP. Participants were classified as receiving “organized screening” if the cost of mammography was covered by the NHIS or other government support. Participants who underwent mammography paid for by private insurance or out-of-pocket expenses were classified as receiving “opportunistic screening.” Screening status was based solely on mammography, as it is the only modality covered by the NCSP. Ultrasound, which is not part of the NCSP and may be provided only following abnormal mammography findings, was not considered in defining screening participation. Screening types were determined based on the most recent screening activity, distinguishing between organized and opportunistic methods.

### Variables

The analysis included age, type of residential area, educational level, and household income. Residential areas were classified as metropolitan or non-metropolitan based on provincial classifications in Korea. Education levels were categorized as middle school or lower, high school, or university or higher. Monthly household income was initially recorded in 13 ranges, from US$0 to US$10,000 or more. These ranges were subsequently grouped into 3 income tiers (low, middle, and high) based on the distribution of participants into income terciles, ensuring a balanced and sufficient distribution within each group. Income group cut-off points were adjusted annually to reflect temporal changes in SES distributions.

### Statistical analysis

Descriptive statistics were used to summarize participant characteristics and breast cancer screening rates by screening type. Organized and opportunistic breast cancer screening rates were calculated and presented as weighted screening rates for each survey year from 2009 to 2021. Trends in screening rates were analyzed using joinpoint regression analysis. Screening rates were fitted to models, which identified the best-fit line, either as a single segment or multiple segments based on observed data patterns over time. Annual percent changes (APCs) for each segment and average annual percent changes (AAPCs) over the 13-year period were calculated along with their respective 95% confidence intervals (CIs).

To examine inequality in breast cancer screening, the slope index of inequality (SII) and the relative index of inequality (RII) were calculated, representing absolute and relative measures of inequality, respectively. These indices quantify gaps in screening rates between the most and least deprived individuals, adjusted for the SES distribution within the population. The SII measures the absolute difference in screening rates between the most and least socioeconomically deprived groups. For SII calculation, social groups were ordered from the most disadvantaged to the most advantaged, with each group assigned a score based on the midpoint of its cumulative population distribution [22]. A positive SII indicates a higher screening rate among individuals in the highest SES group compared to the lowest, whereas a negative SII reflects the opposite [22]. For example, an SII of 10 percentage points (%P) indicates that the highest SES group has a screening rate 10%p higher than the lowest SES group.

The RII represents the ratio of the screening rate of the most deprived group (reference) to the least deprived group, reflecting relative disparities. RII values were computed using multiplicative Poisson regression models, with the exponentiated coefficient (e^β^) representing the relative risk of screening participation across SES groups [23]. An RII greater than 1.0 indicates that the highest SES group has a higher screening rate than the lowest group, whereas an RII less than 1.0 indicates the reverse. An RII equal to 1.0 indicates no inequality; values above 1.0 suggest more favorable outcomes for higher SES groups, while values below 1.0 indicate better outcomes for lower SES groups [24]. For example, an RII of 1.60 means individuals in the highest SES group have a screening rate 1.6 times greater than those in the lowest group [25], whereas an RII of 0.5 indicates the lowest SES group has twice the screening rate of the highest group. Inequality indices were calculated separately by educational and income levels. Estimates of SII and RII were derived from a Poisson regression model adjusted for educational level, income level, age, and residential area. Overall estimates of SII and RII across the 13-year study period were also calculated.

Descriptive analyses and inequality index calculations were conducted using Stata version 16.1 (StataCorp., College Station, TX, USA) and SAS version 9.4 (SAS Institute Inc., Cary, NC, USA). Joinpoint regression analysis was performed using Joinpoint Regression Program version 4.9.1.0 (National Cancer Institute, Rockville, MD, USA).

### Ethics statement

This study was approved by the Institutional Review Board of the National Cancer Center, Korea (IRB approval No. NCC2019-0233). Written informed consent from participants was waived due to the public benefit nature of the study.

## RESULTS

### Characteristics of the study population

The final analysis included 21,974 cancer-free women aged 40-74 who participated in the KNCSS from 2009 to 2021 (Table 1). Approximately 60% to 85% of participants had completed high school, and about 43% to 47% resided in metropolitan areas. Participants were evenly distributed across household income categories due to annual adjustments of income group cut-off points.

### Breast cancer screening trends

**Figure 1.**
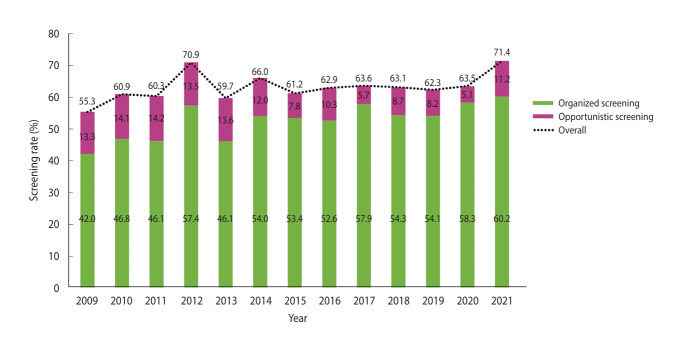
Screening rate for breast cancer according type of screening from 2009 to 2021.

Figure 1 and Supplementary Material 1 illustrate overall breast cancer screening rates and rates by screening type over the 13-year period. The overall screening rate increased from 55.3% in 2009 to 71.4% in 2021. Table 2 presents the overall breast cancer screening rate, which showed an AAPC of 0.7. Overall screening rates were significantly higher among individuals with university or higher education and those in the highest income groups.

Table 3 demonstrates that organized screening rates consistently increased from 42.0% in 2009 to 60.2% in 2021, with a significant AAPC of 1.9. A similar increasing trend was observed across all AAPCs were observed among individuals aged 40-49 years and 60-69 years, residents of non-metropolitan areas, those who had completed high school or university-level education, and participants belonging to the middle income and highest income groups.

Table 4 shows that opportunistic screening rates declined from 13.3% in 2009 to 5.1% in 2020, followed by a sharp increase to 11.2% in 2021. This declining trend was observed across all socioeconomic subgroups. The sharpest decreases were seen among individuals aged 60-69 years, residents of non-metropolitan areas, high school graduates, and those with middle-level income. However, participants with the highest educational and income levels experienced the smallest reductions.

### Educational inequality in breast cancer screening

**Figure 2.**
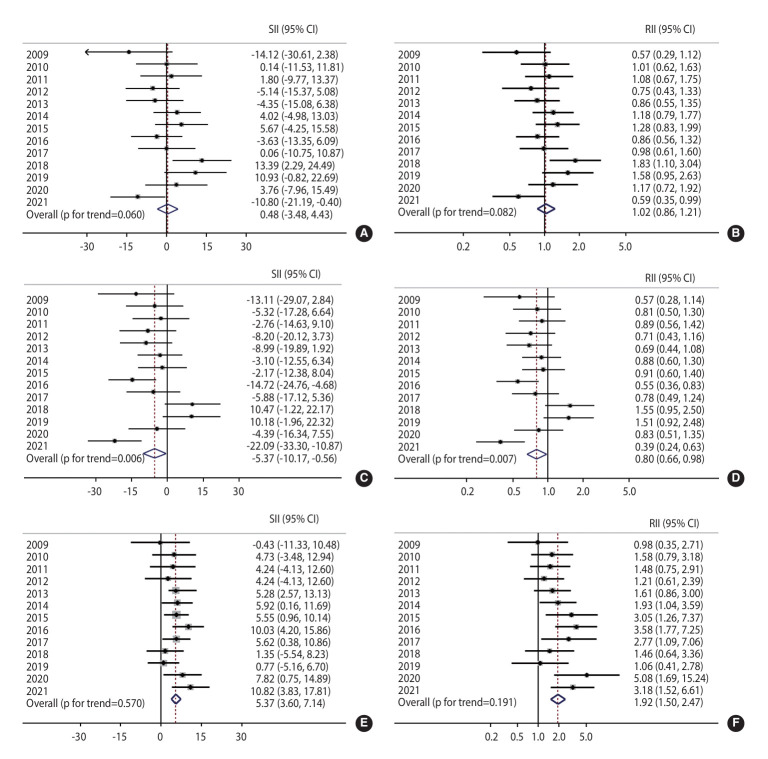
Absolute and relative educational inequalities in overall (A, B), organized (C, D) and opportunistic (E, F) breast cancer screening from 2009 to 2021. SII, slope index of inequality; RII, relative index of inequality; CI, confidence interval.

Absolute and relative educational inequalities in overall, organized, and opportunistic breast cancer screening are presented in Figure 2. For overall screening, no significant absolute or relative educational inequalities were found during the study period, except in 2018. The overall SII was 0.48% (95% CI, -3.48 to 4.43; Figure 2A), and the overall RII was 1.02 (95% CI, 0.86 to 1.21; Figure 2B), indicating the absence of educational inequalities in overall breast cancer screening. For organized screening (Figure 2C and D), significant SII and RII estimates were observed in 2016 and 2021. Negative SII values and RII values below 1.00 indicated higher participation among individuals with lower educational attainment. Specifically, the overall SII for organized screening was -5.37%, and the RII was 0.80 (95% CI, 0.66 to 0.98), demonstrating higher participation rates among less-educated individuals.

For opportunistic screening (Figure 2E and F), positive SII values were consistently observed, resulting in a significant overall SII of 5.37%. This indicates a 5.37%p gap between participants with the highest and lowest educational levels, reflecting persistent educational inequality throughout the 13-year period. Significant absolute inequalities were observed during 2013-2017, 2020, and 2021. Relative inequalities were also evident, with RII values greater than 1.00 in all years except 2009. The overall RII was 1.92, signifying that individuals with higher educational levels were 1.92 times more likely to undergo opportunistic screening.

### Income inequality in breast cancer screening

**Figure 3.**
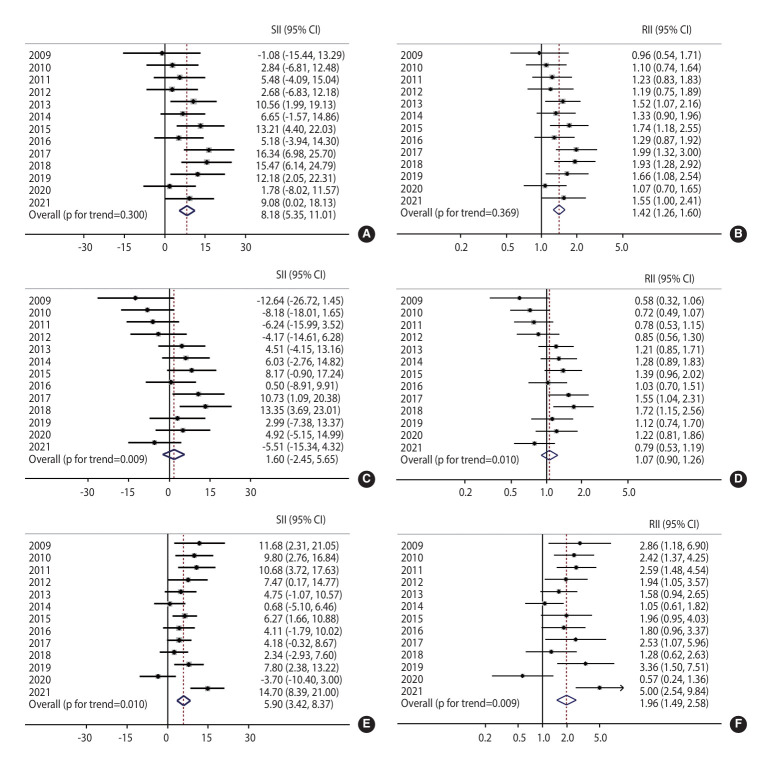
Absolute and relative income inequalities in overall (A, B), organized (C, D) and opportunistic (E, F) breast cancer screening from 2009 to 2021. SII, slope index of inequality; RII, relative index of inequality; CI, confidence interval.

Figure 3 illustrates absolute and relative income inequalities in overall, organized, and opportunistic breast cancer screening. For overall breast cancer screening (Figure 3A and B), significant absolute and relative income inequalities were identified in 2013, 2015, 2017, 2018, 2019, and 2021. The overall SII was 8.18%, and the overall RII was 1.42. Although not significant each year, the inequality indices exhibited a notable pattern. Initially, in 2009, lower-income individuals had higher screening rates; however, from 2010 onwards, screening became increasingly prevalent among higher-income groups.

For organized breast cancer screening (Figure 3C and D), significant absolute and relative income inequalities appeared in 2017 and 2018. The overall SII was 1.60%, and the overall RII was 1.07. No significant income inequalities were observed at the start of the study period; however, from 2013 onward, income inequalities in organized screening began to emerge and increase over time.

For opportunistic breast cancer screening (Figure 3E and F), SII values were predominantly positive throughout the study period, except in 2020. The overall SII was 5.90% over the 13-year period, indicating a 5.90%p gap in screening rates between the highest and lowest income groups. The overall RII was 1.96, indicating that individuals in higher-income groups were 1.96 times more likely to participate in opportunistic screening compared to those in lower-income groups.

## DISCUSSION

This study examined trends in organized and opportunistic breast cancer screening from 2009 to 2021, focusing specifically on socioeconomic inequalities by education and income for each screening type. Our findings demonstrated a significant increasing trend in organized screening participation and a significant decreasing trend in opportunistic screening over time. Women with lower educational levels had higher participation rates in organized breast cancer screening, as evidenced by negative inequality indices. Although income-related inequalities in organized screening were identified in certain years, pooled estimates over the entire study period were not statistically significant in either absolute or relative terms. Conversely, opportunistic screening consistently displayed significant educational and income inequalities throughout the study period.

Previous studies conducted in Switzerland, Spain, and Israel have also reported substantial inequalities in mammography participation, highlighting lower participation among socioeconomically disadvantaged groups [18,26,27]. However, after implementing organized screening programs, participation rates increased, particularly among individuals with lower educational levels [18]. These findings indicate that organized screening programs not only significantly increase overall participation but also effectively reduce inequalities in screening access.

In the current study, we similarly found significant educational inequalities favoring women with lower educational attainment in organized screening contexts. In contrast, opportunistic screening consistently exhibited significant educational inequalities. A study examining 22 European countries reported substantial educational inequalities in breast cancer screening in countries utilizing opportunistic screening approaches (RII, 3.11; 95% CI, 1.78 to 5.42) [17]. However, countries with nationwide organized screening programs, except Finland, showed no educational inequalities. This difference may be attributed to the proactive measures inherent to organized screening programs—such as personalized invitation letters—which can encourage participation even among individuals with lower educational levels.

Additionally, the present study showed no significant pooled estimates for income-related inequalities in organized screening. Similarly, Kim et al. [28] reported a reduction in the relative disparity in breast cancer screening participation between the wealthiest and poorest groups, with screening rates increasing more significantly among lower-income individuals. These findings align with our results, suggesting that organized screening programs contribute to mitigating socioeconomic inequalities in screening participation. Specifically, our results imply that the NCSP in Korea has effectively improved breast cancer screening access for socially vulnerable populations. Overall, the KNCSP seems to have played a critical role in narrowing income-related disparities in screening rates.

A noteworthy finding, however, is the persistent socioeconomic inequality in opportunistic screening observed throughout the study period. This persistence may be explained by the influence of individual preferences, which are strongly shaped by SES. Individuals choosing opportunistic screening may perceive it as offering higher-quality services compared to organized screening [29,30]. Consequently, those in the highest education and income brackets often prefer opportunistic screening through private providers. In Korea, individuals may voluntarily purchase private insurance in addition to the NHIS, which typically covers expenses not reimbursed by NHIS [31]. Private insurance often reimburses opportunistic screening costs, particularly those incurred due to abnormalities detected during screening. Thus, higher SES individuals, more likely to have supplemental private insurance coverage, utilize opportunistic screening more frequently. Conversely, lower-income individuals may avoid opportunistic screening due to its associated out-of-pocket costs. Furthermore, access to opportunistic screening is often geographically limited to urban areas, thereby restricting availability for residents in rural or underserved communities.

Interestingly, substantial fluctuations in opportunistic screening rates occurred between 2019 and 2021, potentially reflecting the impact of COVID-19. During the pandemic, strict social distancing measures and public concerns about infection risk likely influenced screening behaviors. Moreover, disruptions in healthcare services—including temporary suspensions of screening programs and reduced hospital capacity—further limited screening accessibility. However, post-pandemic, opportunistic screening rates rebounded and even surpassed earlier levels. Unlike organized screening, which the government systematically provides and encourages on a predetermined biennial schedule, opportunistic screening relies on individuals’ personal perceptions of need. Consequently, organized screening was relatively stable during the pandemic, while opportunistic screening exhibited greater variability in response to shifting healthcare access and individual preferences.

Although our results indicated no significant pooled estimates of income-related inequality in organized screening, an increasing trend in inequality indices emerged in specific years. This suggests that while the NCSP has effectively reduced inequalities, these disparities have not been entirely eliminated. The introduction of the NCSP has significantly enhanced accessibility to cancer screening by minimizing associated costs, thereby reducing financial barriers and contributing to increased overall screening rates. Nonetheless, fluctuations in income-related inequality highlight the necessity for ongoing efforts to promote equitable participation. Ensuring uniform quality standards across all NCSP-designated screening facilities is crucial, as disparities in facility accessibility, equipment quality, and service levels could discourage participation—particularly among those capable of affording private screening alternatives. Strengthening quality control measures and increasing public awareness of the NCSP may encourage greater participation. Furthermore, structural interventions—such as financial support for follow-up examination costs or introducing paid leave for cancer screening—could further enhance access among lower-income groups.

A prior study in Korea reported absolute socioeconomic inequalities in breast cancer screening over a 10-year period but did not distinguish between screening types [9]. As a result, the specific impact of funding sources—such as the NCSP—on inequalities in screening participation could not be clearly determined. While their findings align with ours regarding overall screening participation, our study specifically found no significant pooled estimates of income-related inequalities in organized screening. By explicitly differentiating screening types and analyzing socioeconomic inequalities accordingly, our results suggest that the NCSP has significantly contributed to reducing inequalities in organized screening. The NCSP provides population-based, government-funded cancer screening services without out-of-pocket expenses, targeting vulnerable populations such as Medical Aid recipients and low-income NHIS beneficiaries. The program utilizes systematic outreach strategies, including mailed invitations and support from public health centers, effectively reducing informational and financial barriers commonly experienced by individuals with lower SES. These structural characteristics of the NCSP likely promote more equitable access and participation, thus helping reduce inequalities in organized screening.

Our study had several limitations. First, data on breast cancer screening experiences were self-reported, potentially introducing recall bias as participants described their previous screening experiences. However, because breast cancer screening in Korea is generally performed biennially and often involves significant healthcare interactions (such as mammography appointments), recall bias is likely minimal. Second, assessing inequalities using the SII and RII methods necessitates hierarchically ordered variables with more than 2 categories; therefore, our analysis was restricted to income and education. Future research should incorporate additional variables to achieve a more comprehensive understanding of inequalities. Lastly, our study did not account for other potential confounders, such as health literacy or geographic accessibility to healthcare facilities, which may influence screening participation.

In conclusion, our findings highlight the beneficial impact of Korea’s organized breast cancer screening program, showing a notable increase in overall participation rates and a reduction in inequalities associated with screening uptake. However, socioeconomic inequalities persist in opportunistic screening and, to a lesser extent, in organized screening contexts, with overall participation rates remaining relatively stagnant. These results indicate the importance of ensuring consistent quality standards across all NCSP-designated screening facilities and implementing supportive policies, such as paid leave for screening and financial assistance for follow-up examinations. Such measures could help mitigate inequalities in cancer screening, lower barriers to participation, and ultimately promote equitable access to preventive healthcare.

## Figures and Tables

**Figure f4-epih-47-e2025031:**
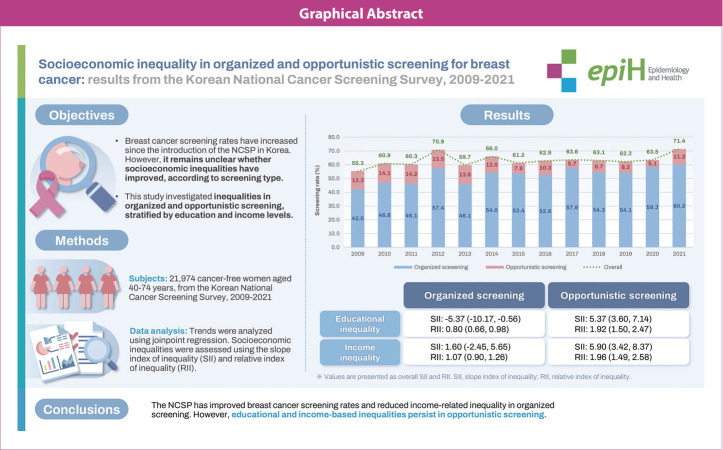


**Table 1.** Distribution of study variables among the participants in the Korean National Cancer Screening Survey for breast cancer, 2009-2021

Variables	2009	2010	2011	2012	2013	2014	2015	2016	2017	2018	2019	2020	2021
Total (n)	834	1,731	1,779	1,762	1,773	1,711	1,711	1,747	1,748	1,754	1,795	1,848	1,781
Age group (yr)													
40-49	41.6	39.8	38.7	38.8	37.5	37.1	36.4	35.6	34.9	34.2	30.6	29.8	31.1
50-59	29.9	31.8	33.4	33.4	34.1	34.2	34.1	34.2	34.1	34.1	31.8	31.2	32.7
60-69	20.4	20.1	19.7	19.6	20.3	20.2	21.1	22.2	23.0	23.8	23.1	24.3	27.5
70-74	8.1	8.3	8.2	8.2	8.1	8.5	8.4	8.0	8.0	7.9	14.5	14.7	8.7
Residential area													
Metropolitan	46.6	44.5	45.5	44.9	44.6	45.2	46.1	44.6	45.5	44.9	44.4	46.1	43.9
Non-metropolitan	53.4	55.5	54.5	55.1	55.4	54.8	53.9	55.4	54.5	55.1	55.6	53.9	56.1
Educational level													
Middle school or lower	39.8	30.0	28.8	26.6	17.9	17.3	19.5	16.2	19.4	16.7	19.7	19.0	15.0
High school	45.7	54.2	54.7	57.5	59.2	57.7	59.0	56.2	55.3	58.1	57.2	57.2	55.2
University or higher	14.5	15.8	16.5	15.9	22.9	25.0	21.5	27.6	25.3	25.2	23.1	23.8	29.8
Household income													
Low	30.7	34.8	33.9	39.8	36.5	38.3	37.4	39.4	36.0	36.1	34.4	36.1	31.8
Middle	42.5	35.0	35.5	32.5	31.8	36.3	39.2	40.4	42.4	36.8	35.7	31.6	31.1
High	26.8	30.2	30.6	27.7	31.7	25.4	23.4	20.2	21.6	27.1	29.9	32.3	37.1

Values are presented as %.

**Table 2.** Overall screening rates (%) for breast cancer according to socioeconomic status in the Korean National Cancer Screening Survey, 2009-2021

Variables	2009	2010	2011	2012	2013	2014	2015	2016	2017	2018	2019	2020	2021	AAPC (95% CI)
Total	55.3	60.9	60.3	70.9	59.7	66.0	61.2	62.9	63.6	63.1	62.3	63.5	71.4	0.7 (-0.3, 1.7)
Age group (yr)														
40-49	51.3	59.3	57.3	69.2	63.8	66.1	62.3	58.2	63.9	65.9	66.8	64.1	68.9	1.0 (-0.4, 2.3)
50-59	58.0	66.7	66.7	77.5	59.2	70.0	64.6	62.2	64.9	66.7	64.6	65.4	75.8	0.2 (-1.2, 1.8)
60-69	66.1	58.7	58.1	68.0	56.5	63.5	56.9	70.0	64.6	61.0	61.4	66.6	72.2	1.0 (-1.0, 3.5)
70-74	39.0	51.4	54.0	59.3	50.8	55.7	53.8	67.5	54.2	41.0	49.3	52.9	61.5	0.3 (-1.5, 2.5)
Residential area														
Metropolitan	54.6	65.1	63.8	67.8	64.1	68.6	60.0	63.6	64.9	65.6	65.6	61.7	71.2	0.4 (-0.5, 1.5)
Non-metropolitan	55.9	57.5	57.5	73.5	56.1	63.9	62.2	62.4	62.6	61.0	59.7	65.0	71.6	0.7 (-0.6, 2.3)
Educational level														
Middle school or lower	60.5	59.3	58.7	66.8	53.4	63.4	53.3	66.7	61.6	47.1	51.6	57.5	69.4	-0.0 (-2.7, 2.2)
High school	50.5	61.2	60.9	74.9	61.6	66.1	63.2	62.7	62.9	65.2	63.2	64.9	73.4	0.6 (-0.5, 1.8)
University or higher	56.2	62.9	61.2	63.2	59.6	67.8	63.0	61.2	66.6	68.7	69.3	64.6	68.7	1.1 (0.1, 2.3)^1^
Household income														
Low	59.4	60.1	59.4	70.0	57.8	62.2	56.9	63.6	60.8	55.2	56.4	60.5	69.3	0.0 (-1.5, 1.6)
Middle	52.1	59.0	58.3	71.7	56.4	69.9	61.1	60.6	60.5	64.8	60.5	66.1	71.1	0.8 (-0.7, 2.6)
High	55.7	63.9	63.8	71.3	65.2	66.2	68.3	66.3	74.4	71.1	71.3	64.1	73.5	1.0 (0.2, 1.9)^1^

AAPC, average annual percent change; CI, confidence interval.

1 AAPC is significantly different from zero at the alpha=0.05 level.

**Table 3.** Organized screening rates (%) for breast cancer according to socioeconomic status in the Korean National Cancer Screening Survey, 2009-2021

Variables	2009	2010	2011	2012	2013	2014	2015	2016	2017	2018	2019	2020	2021	AAPC (95% CI)
Total	42.0	46.8	46.1	57.4	46.1	54.0	53.4	52.6	57.9	54.3	54.1	58.3	60.2	1.9 (0.7, 3.4)^1^
Age group (yr)														
40-49	36.0	42.0	39.4	54.5	46.8	52.0	54.3	45.4	59.1	55.6	55.7	56.2	53.7	4.0 (1.7, 6.5)^1^
50-59	44.8	53.6	53.1	64.2	47.6	58.9	55.6	53.6	57.7	57.5	56.8	60.1	63.6	1.1 (-0.1, 2.4)
60-69	53.9	48.6	48.6	53.1	45.9	53.3	51.8	61.0	58.6	54.0	55.1	63.1	64.8	2.3 (1.0, 4.0)^1^
70-74	32.9	39.7	43.3	53.7	37.0	45.0	45.1	57.6	51.2	46.0	43.6	51.0	56.4	1.7 (-1.1, 5.6)
Residential area														
Metropolitan	40.5	49.0	47.9	52.9	50.4	58.1	53.9	51.7	58.2	57.4	58.7	56.2	58.5	1.8 (0.7, 3.2)^1^
Non-metropolitan	43.4	45.0	44.6	61.1	42.6	50.7	53.0	53.3	57.6	51.8	50.5	60.1	61.6	2.0 (0.0, 4.3)^1^
Educational level														
Middle school or lower	49.4	49.3	49.2	57.0	42.8	53.3	47.9	59.0	58.1	39.2	45.3	55.6	64.6	1.2 (-1.4, 3.6)
High school	36.7	46.5	45.8	60.0	48.5	55.3	56.2	54.6	57.4	57.6	55.7	60.4	64.4	2.3 (0.6, 4.2)^1^
University or higher	38.8	43.1	41.7	48.8	42.4	51.8	50.8	44.8	58.9	56.7	57.9	55.5	50.5	2.0 (0.7, 5.0)^1^
Household income														
Low	50.8	50.3	49.7	59.4	45.3	50.9	50.5	55.4	56.4	47.7	51.1	56.7	64.6	1.3 (-0.1, 2.8)
Middle	38.6	45.5	44.7	57.8	44.5	57.8	54.2	51.2	55.5	56.0	52.7	60.0	61.0	2.2 (0.6, 4.1)^1^
High	37.5	44.4	43.7	54.2	48.6	53.5	56.9	50.1	65.1	60.8	59.3	58.5	55.9	2.6 (1.4, 4.6)^1^

AAPC, average annual percent change; CI, confidence interval.

1 AAPC is significantly different from zero at the alpha=0.05 level.

**Table 4.** Opportunistic screening rates (%) for breast cancer according to socioeconomic status in the Korean National Cancer Screening Survey, 2009-2021

Variables	2009	2010	2011	2012	2013	2014	2015	2016	2017	2018	2019	2020	2021	AAPC (95% CI)
Total	13.3	14.1	14.2	13.5	13.6	12.0	7.8	10.3	5.7	8.7	8.2	5.1	11.2	-5.4 (-8.7, -2.3)^1^
Age group (yr)														
40-49	15.3	17.3	18.0	14.6	17.0	14.2	8.0	12.8	4.8	10.3	11.1	7.9	15.2	-4.4 (-8.5, -1.0)^1^
50-59	13.2	13.1	13.6	13.2	11.6	11.0	9.0	8.7	7.2	9.2	7.9	5.3	12.2	-2.6 (-6.5, -0.3)^1^
60-69	12.2	10.1	9.5	14.9	10.6	10.1	5.1	9.0	5.9	7.0	6.3	3.6	7.4	-6.6 (-11.6, -2.0)^1^
70-74	6.0	11.7	10.7	5.6	13.8	10.7	8.7	9.9	3.0	5.0	5.8	1.9	5.1	-8.6 (-18.1, 0.4)
Residential area														
Metropolitan	14.1	16.1	15.9	14.8	13.7	10.5	6.1	11.9	6.6	8.1	6.9	5.5	12.7	-3.8 (-8.2, -1.1)^1^
Non-metropolitan	12.6	12.5	12.8	12.4	13.6	13.2	9.2	9.1	4.9	9.2	9.2	4.8	10.0	-4.8 (-8.3, -1.7)^1^
Educational level														
Middle school or lower	11.2	10.0	9.5	9.8	10.6	10.1	5.4	7.7	3.5	7.8	6.2	1.9	4.9	-7.2 (-11.5, -3.9)^1^
High school	13.8	14.7	15.1	15.0	13.1	10.8	6.9	8.1	5.6	7.6	7.6	4.5	9.1	-7.9 (-12.5, -4.2)^1^
University or higher	17.4	19.7	19.5	14.4	17.3	16.0	12.3	16.4	7.7	12.0	11.4	9.2	18.3	-1.8 (-5.5, 1.1)
Household income														
Low	8.6	9.9	9.7	10.6	12.5	11.3	6.4	8.2	4.5	7.4	5.3	3.8	4.8	-6.3 (-10.5, -2.3)^1^
Middle	13.5	13.5	13.6	13.9	11.9	12.2	6.9	9.4	5.0	8.8	7.8	6.1	10.1	-5.8 (-9.2, -2.7)^1^
High	18.2	19.6	20.1	17.1	16.6	12.7	11.4	16.2	9.3	10.3	12.0	5.7	17.6	-2.5 (-7.6, 0.7)

AAPC, average annual percent change; CI, confidence interval.

1 AAPC is significantly different from zero at the alpha=0.05 level.

## Supplementary materials

Supplementary Material 1. Trends in breast cancer screening from 2009 to 2021 by types of screening, (A) – Overall; (B) – Organized; (C) – Opportunistic;
