# Supplementary material for: Socioeconomic inequality in organized and opportunistic screening for breast cancer: results from the Korean National Cancer Screening Survey, 2009-2021
Source: Epidemiol Health. 2025 May 30;47:e2025031. doi: 10.4178/epih.e2025031 (PMC12425860; doi:10.4178/epih.e2025031)
Supplement: Supplementary Material 1. — Trends in breast cancer screening from 2009 to 2021 by types of screening, (A) – Overall; (B) – Organized; (C) – Opportunistic; [file epih-47-e2025031-Supplementary-1.pptx]

## Slide 1
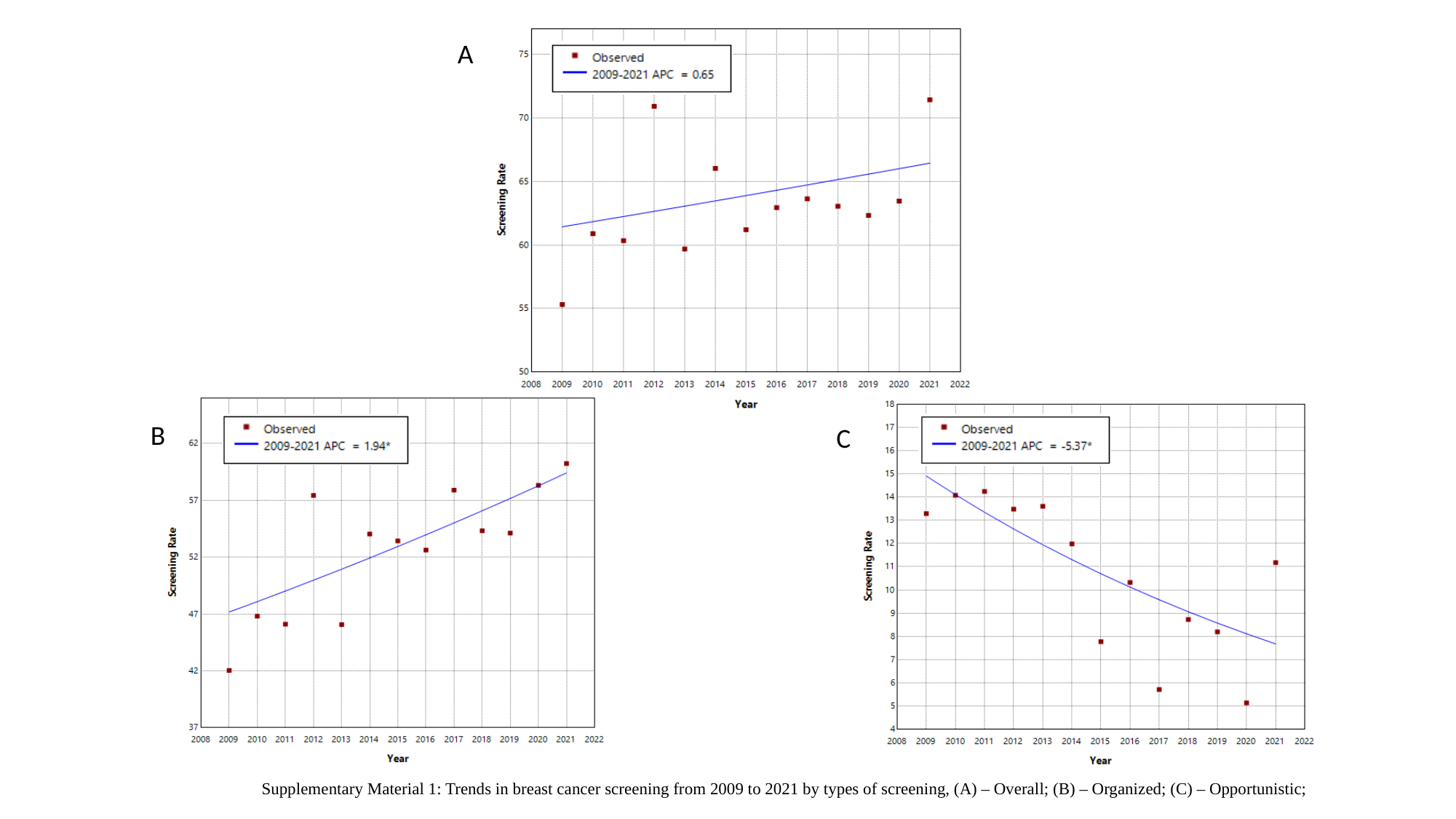

A
B
C
Supplementary Material 1: Trends in breast cancer screening from 2009 to 2021 by types of screening, (A) – Overall; (B) – Organized; (C) – Opportunistic;
